# Pembrolizumab in a Patient With a Metastatic CASTLE Tumor of the Parotid

**DOI:** 10.3389/fonc.2019.00734

**Published:** 2019-08-14

**Authors:** Lisa Lorenz, Joscha von Rappard, Walter Arnold, Nicole Mutter, Udo Schirp, Andreas Scherr, Andreas Werner Jehle

**Affiliations:** ^1^Department of Internal Medicine, Hirslanden Klinik St. Anna, Lucerne, Switzerland; ^2^Department of Pathology, Cantonal Hospital Lucerne, Lucerne, Switzerland; ^3^Department of Oncology, Hirslanden Klinik St. Anna, Lucerne, Switzerland; ^4^Institute of Radiology and Nuclear Medicine, Hirslanden Klinik St. Anna, Lucerne, Switzerland; ^5^Department of Pneumology, Hirslanden Klinik St. Anna, Lucerne, Switzerland; ^6^Transplantation Immunology and Nephrology, University Hospital Basel, Basel, Switzerland

**Keywords:** CASTLE, parotid, extrathyroidal, carcinoma, thymus-like, PD-L1, immunotherapy, checkpoint inhibitor

## Abstract

Carcinoma showing thymus-like elements (CASTLE) is a rare tumor, most commonly found in the thyroid gland. Here we report a case of CASTLE tumor localized to the parotid gland, recognized in retrospect after a late manifestation of symptomatic pleural carcinomatosis. The original tumor in the parotid gland was treated by surgery followed by radiotherapy. Ten years later, a metastatic disease with recurrent pleural effusions occurred. Pleural carcinomatosis was strongly positive for CD5, CD117, and p63 as was the original tumor of the parotid, which allowed the diagnosis of a CASTLE tumor. Additionally, the pleural tumor expressed high levels of programmed death ligand 1 (PD-L1), and the patient underwent treatment with the monoclonal PD-L1 inhibitor pembrolizumab achieving a partial remission. To the best of our knowledge, this is the first patient with a metastatic CASTLE tumor treated with a PD-L1 inhibitor.

## Background

Carcinoma showing thymus-like differentiation (CASTLE) is a rare tumor usually of the thyroid gland or adjacent soft tissues of the neck (1). The entity was first described as an intrathyroidal epithelial thymoma by Miyauchi et al. (2) and later renamed as CASTLE by Chan and Rosai, who proposed that these tumors arise either from ectopic thymuses or from remnants of the branchial pouches that differentiate along the thymic line (3).

CASTLE tumors show not only structural similarity to thymic tissue but also express molecular markers of thymomas and thymic carcinomas (4, 5). In immunohistochemical staining, they are positive for CD5 in nearly all cases and positive for CD117 and p63, but negative for markers of thyroid carcinomas, such as thyroglobulin and calcitonin (5).

CASTLE tumors are indolent and slow-growing malignant neoplasms. In about 30–50% of cases, that describe the nodal status, lymph node involvement is reported and in 14–29% metastatic disease (6). Radical surgery is the treatment of choice for CASTLE, and radiotherapy is considered in patients with positive lymph node status. For advanced and metastatic disease, the standard treatment is chemotherapy (6). However, because of the rarity of CASTLE, data on the effectiveness of adjuvant radiotherapy and chemotherapy is missing.

Immunotherapy has become a cornerstone in the treatment of several malignancies in the past few years and involves the use of immune checkpoint inhibitors including antibodies against programmed cell death protein 1 (PD-1), and programmed death ligand 1 (PD-L1) (7). When PD-1, which is primarily expressed on activated T cells, binds PD-L1 on tumor cells, the cytotoxic T-cell response is reduced. PD-L1 expression is associated with a better response to anti-PD-1 treatment in non-small cell lung cancer (8, 9) as well as in thymic carcinoma (10). Herein, we report what we believe is the first case of a metastatic CASTLE tumor systemically treated with the PD-L1 inhibitor pembrolizumab.

## Case Presentation

A 79-year old woman was hospitalized on April 25, 2018, at which time she had progressive dyspnea over the past 5 weeks, productive cough, poor appetite, and weight loss of 5 kg over the last 6 months currently weighing 81 kg. Her medical history included an invasive ductal breast cancer pT1cN0M0 G2 ER 90%, PR 20% 20 years ago treated with lumpectomy, radiotherapy, and tamoxifen until 2004, and an undifferentiated cancer of the right parotid pT1 (17 mm) pN2 (1/2 positive in level I, 1/11 positive in level II, no extranodal extensions) pM0 diagnosed 2008 and treated with a total parotidectomy achieving R0 margins and a selective neck dissection levels II-IV followed by radiotherapy.

**Figure 1:**
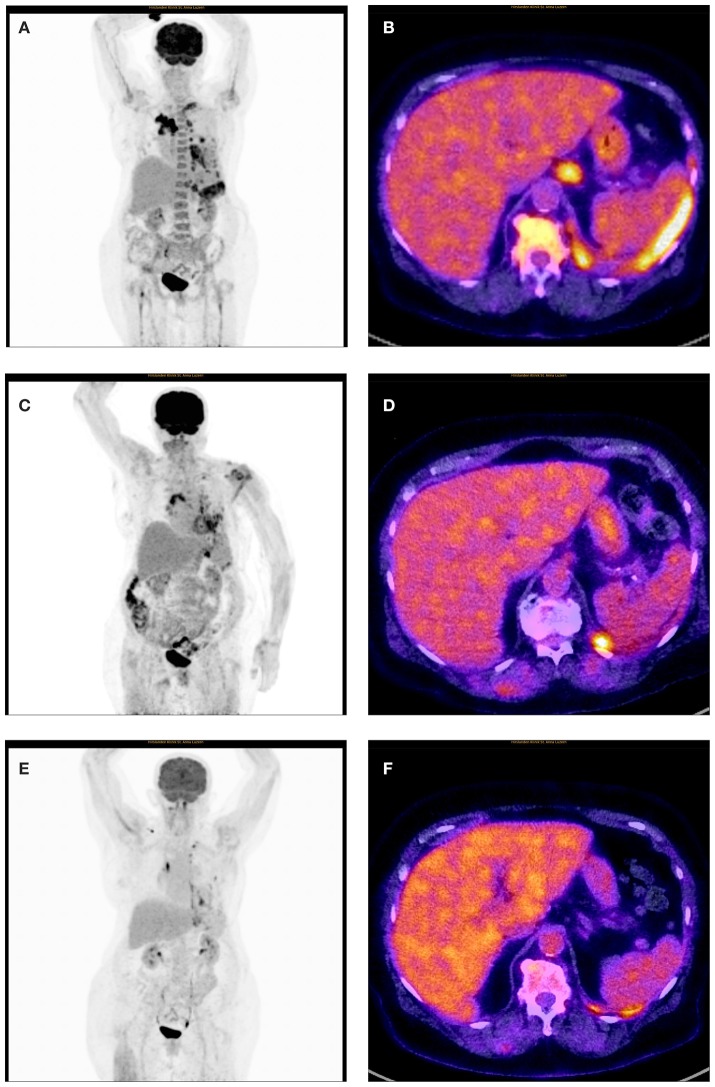
Initial and follow-up FDG PET/CT: **(A)** Initial FDG-MIP with multiple hypermetabolic lesions in the upper lobe of the right lung, in mediastinal, hilar and upper mesenteric lymph nodes and at the left pleura. Increased turnover of the bone marrow. **(B)** Initial fusion FDG-PET/CT axial slice with pathologic high FDG-uptake in the left pleura, upper mesenteric lymph nodes, and bone marrow. Normal presentation of liver, spleen, and stomach. **(C)** Follow-up FDG-MIP after 4 months on pembrolizumab shows a generally reduced metabolism at all tumor locations. Normalization of the bone marrow turnover. Due to newly incipient traumatic injury at the left shoulder the patient could not lift up the left arm. **(D)** Fusion FDG-PET/CT axial slice after 4 months on pembrolizumab with residual FDG-uptake at the left pleura and in upper mesenteric lymph nodes. Normal presentation of the bone marrow. **(E)** Follow-up FDG-MIP after 11 months on pembrolizumab shows further regression of lesions in the upper lobe of the right lung as well as in mediastinal, hilar, and upper mesenteric lymph nodes (the focal lesions in the left mediastinum and supraclavicular right represent the central venous catheter). Residual focal FDG-Uptake in the left pleura. Normal turnover of the bone marrow. **(F)** Fusion FDG-PET/CT axial slice after 11 months on pembrolizumab with only little elevated FDG-uptake in the left pleura. The formerly upper mesenteric lymph node is healed up. Normal presentation of liver, spleen, stomach, and bone marrow.

**Figure 2:**
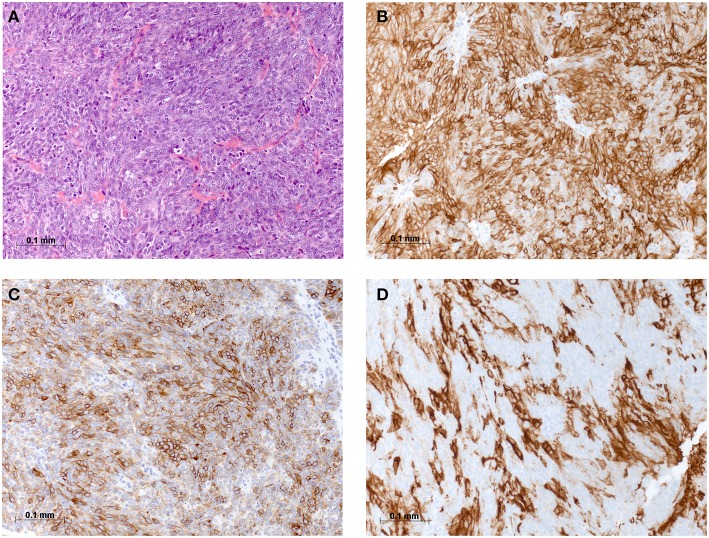
Histomorphological and immunohistochemical analysis of parietal pleura, biopsy from 2018: **(A)** H & E staining, **(B)** Immunostaining for CD5, **(C)** Immunostaining for CD117, and **(D)** Immunostaining for PD-L1.

**Figure 3:**
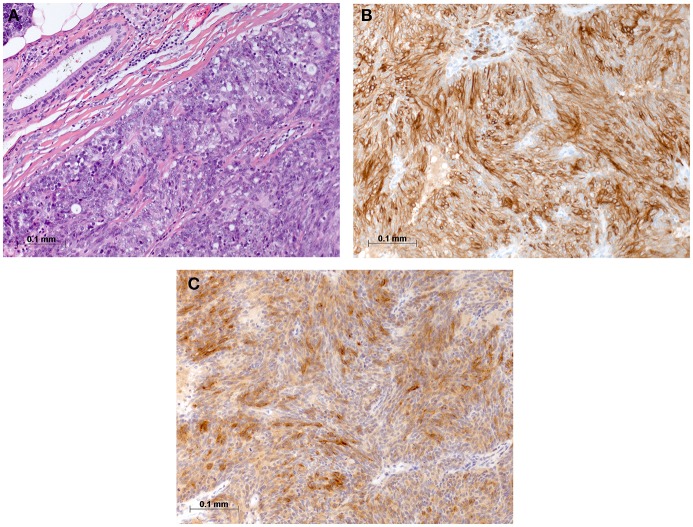
Histomorphological and immunohistochemical analysis of parotid, tumor excisate from 2008: **(A)** H & E staining, **(B)** Immunostaining for CD5, and **(C)** Immunostaining for CD117.

On physical examination, the patient was in mild respiratory distress. The pulmonary exam revealed dullness to percussion, decreased fremitus, and decreased breathing sounds in up to two-thirds of the left lung field. Oxygen saturation was 92% with the patient breathing ambient air, and arterial blood gases showed partial respiratory insufficiency. A chest X-ray confirmed a left-sided pleural effusion, and thoracentesis removed 1.5 liters of turbid fluid. Biochemical analysis of the fluid established an exudative etiology. Atypical cells with immunocytochemical positivity for p40, cytokeratin 5, and p63 in the cytopathology specimen were primarily suggestive of a non-small cell lung cancer, and a CT scan of the thorax showed a mass (4.2 × 2.2 × 2.8 cm) in the right upper lobe and extensive cardiophrenic, mediastinal, and hilar lymphadenopathy as well as enlarged lymph nodes along the left arteria mammaria interna. An FDG PET/CT scan confirmed these findings, and the scan revealed a hypermetabolic activity in mesenteric lymph nodes and at the left pleura (Figures 1A,B). Thoracoscopy and biopsy of the parietal pleura were performed. Surprisingly, histopathological examination revealed a non-small cell carcinoma (Figure 2A) expressing CD5 (Figure 2B), CD117 (Figure 2C), and p63 all indicating a carcinoma of thymic origin (5). With no signs of a thymic pathology, the histopathological diagnosis of CASTLE was established, and as additional immunostainings for CD5 (Figure 3B) and CD117 (Figure 3C) in the specimen of the parotid cancer diagnosed 2008 were positive also, a rare case of a CASTLE tumor of the parotid healed at the primary and neck sites, but with late metastatic dissemination was diagnosed.

As CD117 was highly overexpressed in the tumor, exons 9, 11, 13, 14, and 17 of the C-KIT gene were analyzed after extraction of genomic DNA by PCR using specific primers, but no common mutation was found consistent with previous reports (11, 12). Anticancer immunity and potential response to immunotherapy were assessed by PD-L1 staining of the pleural tumor, which showed a high expression level of 60% (Figure 2D).

The patient underwent talc pleurodesis and left partial pleurectomy for recurrent pleural effusions. Systemically, disseminated metastatic disease was treated with the monoclonal PD-L1 inhibitor pembrolizumab at a dose 200 mg every 3 weeks. Four months after the start of immunotherapy a follow-up FDG PET/CT study showed a partial remission with a moderate morphologic reduction of the tumor load, but a markedly reduced metabolic activity at all tumor sites (Figures 1C,D). Pembrolizumab treatment continued at a dose of 200 mg IV every 3 weeks. The patient showed a mild skin rash treated with low dose prednisolone (20 mg with tapering) for 2 weeks. No other adverse events associated with pembrolizumab were observed, and the therapy was tolerated well. At the last follow-up, the patient was in good clinical condition and had gained 3 kg of weight. On March 28th, 2019, a second follow-up FDG PET/CT showed no residual pleural effusion (not shown) and a further regression of lesions in the right lung and lymph nodes. Residual focal FDG-Uptake was seen in the left pleura (Figures 1E,F).

## Discussion

CASTLE is a rare malignant tumor, and in the majority of cases, the tumor is localized in the thyroid gland (6). Histologically, CASTLE resembles thymic carcinoma, and the current postulation is that these tumors arise from remnants of the branchial pouches with thymic differentiation, or ectopic thymuses (3, 13). This hypothesis is further supported by the observation that these tumors show positive immunostaining for CD5, CD117, and p63 reminiscent of thymomas and thymic carcinomas (4, 5). As Gao et al. (6) suggested the low number of reported cases of this disease may be related to the variable, and non-specific clinical manifestation and a conclusive diagnosis can only be maid by postsurgical pathological examination including immunohistochemical studies. A few extrathyroidal cases of CASTLE have been reported, and one of them localized to the right parotid and was classified as WHO stage III, pT1pN1cM0, and expressed CD5, CD117, P40, and P63 (14). The patient was treated with right total parotidectomy and ipsilateral selective neck dissection followed by adjuvant radiotherapy and remained disease free for more than 1 year after completing treatment on follow-up with MRI scan (14). In the case reported here, the diagnosis of the primary tumor in the parotid was made in retrospect after the disease was discovered in the pleural biopsy and after the parotid specimen was reanalyzed with additional staining for CD5 and CD117. We can not exclude the possibility of a metachronous CASTLE, but this seems unlikely due to the rarity of the disease. Also, we can not exclude with certainty that the lesion in the right upper lobe was a primary lung cancer with metastasis of the collateral, left-sided pleura. But, this alternative diagnosis seems less likely in this non-smoking patient presenting with weight loss over 6 months and dyspnea due to the process in the left pleura with large, left-sided pleural effusion. Expression of CD5 and CD117 strongly support CASTLE with metastatic dissemination, though a minority of NSCLC are known to express CD5 and CD117 (15).

The parotid tumor pT1pN2pM0 diagnosed 2008 was treated as undifferentiated cancer with a total parotidectomy and a selective neck dissection levels II-IV followed by radiotherapy, a therapeutic approach typically used for CASTLE as well.

Chemotherapeutic regimens for advanced, metastatic CASTLE have been used based on those designed for thymic carcinomas (16) with the assumption that the tumor biology of CASTLE is similar to thymic carcinomas, but available data is very limited, and the benefit for patient survival unknown (6). For metastatic thymic carcinomas, the response rate to chemotherapy is <50% (17), and new effective therapies are awaited. Importantly, thymic carcinomas express high levels of PD-L1 (18), and recently, a first prospective study with the anti-PD-L1 antibody pembrolizumab in patients with no response to chemotherapy or recurrent, progressive disease was encouraging showing a positive correlation between response, progression-free survival, overall survival, and PD-L1 expression (10). To the best of our knowledge, expression levels of PD-L1 in CASTLE has not been investigated. In our patient 60% of tumor cells expressed PD-L1, and after intensive discussion in our interdisciplinary tumor board, a first line systemic therapy with pembrolizumab was initiated, which resulted in a partial response documented in a follow-up FDG PET/CT at 4 months. Except a skin rash no other adverse event potentially related to pembrolizumab was observed. At the last follow-up in March 2019 the patient was in good condition and a second follow-up FDG-PET/CT showed a sustained partial response.

CASTLE tumors may be found also in the parotid, have a slow and indolent course in spite of their undifferentiated histopathology, express PD-L1 and are therefore good targets for complementary immunotherapy when needed for recurrent/metastatic disease.

## Ethics Statement

We received a written informed consent of the patient for the publication of this case report.

## Author Contributions

LL collected all data. LL and JvR wrote the first draft of the manuscript. WA performed histological analyses, provided images, and reviewed the manuscript. NM treated the patient and reviewed the manuscript. US provided FDG PET/CT images and reviewed the manuscript. AS reviewed the manuscript. AJ wrote the manuscript. All authors read and approved the submitted version of the manuscript.

### Conflict of Interest Statement

The authors declare that the research was conducted in the absence of any commercial or financial relationships that could be construed as a potential conflict of interest.
